# A single-institution study evaluating the fertility of young adults with malignancy treated with immunotherapy

**DOI:** 10.1093/oncolo/oyaf310

**Published:** 2025-10-01

**Authors:** Jenny D Gong, Kathryn Coyne, Matthew Mirsky, Katherine Daunov, Rebecca Flyckt, Iris Y Sheng

**Affiliations:** Department of Medicine, University Hospitals Cleveland Medical Center, Cleveland, OH 44106, United States; Department of Oncofertility, University Hospitals Cleveland Medical Center, Cleveland, OH 44106, United States; Department of Hematology/Oncology, University Hospitals Cleveland Medical Center, Cleveland, OH 44106, United States; Department of Oncofertility, University Hospitals Cleveland Medical Center, Cleveland, OH 44106, United States; Department of Oncofertility, University Hospitals Cleveland Medical Center, Cleveland, OH 44106, United States; Department of Hematology/Oncology, University Hospitals Cleveland Medical Center, Cleveland, OH 44106, United States

**Keywords:** immunotherapy, fertility, reproduction, pregnancy, cancer

## Abstract

Immune checkpoint inhibitors (ICIs) are increasingly used among multiple types of cancers, but there are limited studies on the long-term effects of ICIs on fertility. Our study examines reproductive outcomes following ICI treatment and details experiences with fertility services at a single tertiary institution. A total of 184 female patients between the ages of 18-45 with documented diagnoses of malignancies were treated with ICIs from January 2012 to December 2023 at a single tertiary medical center. Demographics, oncologic, and fertility data were collected. Of 184 patients, 68 (37.0%) had documented fertility discussions prior to initiation of ICIs and 36 (19.6%) patients were referred to reproductive medicine. Of the referred patients, the median age at time of ICI therapy was 32 years. 27 (75%) were Caucasian and 6 (16.7%) were African American. The most common cancers were breast adenocarcinoma (52.8%), hematologic malignancies (25%), and melanoma (13.9%). 21 (58.3%) underwent a fertility preservation cycle (oocyte or embryo cryopreservation). There were 3 individuals with successful pregnancies. One individual used in vitro fertilization to conceive, resulting in 2 full-term live births. The other two conceived via natural conception, resulting in one full-term live birth each. Time to conception ranged from 12-27 months following ICI completion. All 3 patients underwent treatment with nivolumab, 2 of whom received chemotherapy treatment beforehand. Successful conception following treatment with immunotherapy is possible. Larger multi-institutional studies are needed to further delineate the long-term effect of ICIs on fertility.

## Discussion

Immune checkpoint inhibitors (ICIs) are the standard of care for the treatment of multiple types of cancers. While they have led to dramatic and durable responses, ICIs carry the risk of multi-organ immune-related adverse events, including in the gonads. In mouse models, blockade of the PD1-PDL1 axis has been shown to deplete ovarian reserve which may lead to permanent effects on fertility.^1^ Unfortunately, there are limited studies on potential reproductive toxicities and the long-term effects on fertility in humans. Thus, it is important to characterize the cytotoxic effect and duration of ICIs on fertility.

Our study examined reproductive outcomes after treatment with ICIs and referral rates to our oncofertility program. A total of 184 female patients between the ages of 18-45 years with documented diagnoses of malignancies were treated with ICIs from January 2012 to December 2023 at a single tertiary medical center in Cleveland. Two patients were excluded due to limited data. Demographics, oncologic, documented fertility discussions, and reproductive data (prior pregnancies, number of pregnancies, fertility preservation method, mode of pregnancy, and duration until pregnancy) were collected. Fertility discussions were defined as any mention of reproductive planning or referrals to oncofertility prior to ICI initiation. Successful conception was identified as a positive pregnancy test or through documentation in provider notes. Human subject approval was obtained at University Hospitals Cleveland Medical Center (STUDY20221434).

Of 184 patients, 68 (37.0%) had documented fertility discussions prior to ICI start and 36 (19.6%) patients were referred to oncofertility (Table 1). Of the referred patients, the median age at time of ICI therapy was 32 years. The majority of patients were Caucasian (75%). The most common cancers were breast adenocarcinoma (52.8%), hematologic malignancies (25%), and melanoma (13.9%). Twenty patients (55.6%) received concurrent chemotherapy and ICIs, 10 patients (27.8%) received chemotherapy prior to ICIs, and 6 patients (16.7%) received ICIs alone. Twenty-one (58.3%) underwent a fertility preservation cycle (oocyte or embryo cryopreservation). One received ovarian stimulation, but cycle cancellation was recommended due to poor response and oocyte retrieval was cancelled.

**Table 1.** Demographics of young adults referred to oncofertility providers prior to administration of ICIs.

N	36
**Median age at administration of immunotherapy**	32 years
**Sex**	
** -Female**	36/36 (100%)
**Race**	
** -White**	27/36 (75%)
** -Black/African American**	6/36 (16.7%)
** -Other**	3/36 (8.3%)
**Ethnicity**	
** -Hispanic and/or Latino**	1/36 (2.8%)
** -Non-Hispanic/Latino**	35/36 (97.2%)
**Type of cancer**	
** -Breast adenocarcinoma**	19/36 (52.8%)
** -Hematologic malignancy**	9/36 (25%)
** -Melanoma**	5/36 (13.9%)
** -CNS malignancy**	1/36 (2.8%)
** -GI malignancy**	1/36 (2.8%)
** -Lung malignancy**	1/36 (2.8%)
**Cancer stage at time of diagnosis**	
** -Stage I**	2/36 (5.6%)
** -Stage II**	10/36 (27.8%)
** -Stage III**	15/36 (41.7%)
** -Stage IV**	8/36 (22.2%)
** -Missing**	1/36 (2.8%)
**Immunotherapy received**	
** -Pembrolizumab**	26/36 (72.2%)
** -Nivolumab**	8/36 (22.2%)
** -Ipilimumab**	3/36 (8.3%)
** -Durvalumab**	2/36 (5.6%)
** -Atezolizumab**	1/36 (2.8%)
**Timing of immunotherapy treatment**	
** -Combination chemotherapy and ICI**	20/36 (55.6%)
** -Chemotherapy prior to ICI**	10/36 (27.8%)
** -ICI alone**	6/36 (16.7%)
**Fertility preservation method**	
** -Oocyte cryopreservation**	18/36 (50.0%)
** -Embryo cryopreservation**	3/36 (8.3%)
** -Ovarian stimulation only**	1/36 (2.8%)
** -Declined preservation**	14/36 (38.9%)
**Successful conception after immunotherapy**	3/36 (8.3 %)

ICI, immune checkpoint inhibitor; CNS, central nervous system; GI, gastrointestinal.

There were 3 individuals with successful pregnancies, all of whom were referred to oncofertility (Table 2). One individual conceived via in vitro fertilization (IVF)/frozen embryo transfer (FET) with utilization of embryos frozen prior to ICI treatment, resulting in 2 separate full-term live births. The other two conceived via natural conception, resulting in one full-term live birth each. Time to conception ranged from 12-27 months following ICI completion. All 3 patients underwent treatment with nivolumab, 2 of whom received chemotherapy prior.

**Table 2.** Reproductive data for individuals with successful conception following ICI treatment.

Patient number	1	2	3
**Age at time of ICI**	24	22	21
**Diagnosis**	Hodgkin lymphoma	Melanoma	Hodgkin lymphoma
**Stage**	IV	IIIA	IIA
**ICI received**	Nivolumab	Nivolumab	Nivolumab
**Number of pregnancies prior to ICI**	0	0	1
**Number of pregnancies following ICI**	2	1	1
**Number of successful pregnancies following ICI**	2	1	1
**Fertility preservation method**	Embryo cryopreservation	Declined	Oocyte cryopreservation
**Mode of pregnancy**	IVF via FET	Natural	Natural
**Duration after treatment until pregnancy (mo)**	12	15	27

ICI, immune checkpoint inhibitor; IVF, in vitro fertilization; FET, frozen embryo transfer.

Early discussions regarding fertility preservation prior to ICI treatment are necessary. In our center, only 37% of patients were counseled on fertility preservation methods. Increasing provider education, utilizing order sets, and implementing oncofertility navigators have been shown to double utilization of services.^2^^,^^3^ There is a misconception that fertility preservation may delay cancer treatment. While ovarian stimulation alone can take around 2-5 weeks, alternatives such as ovarian tissue cryopreservation or gonadotropin-releasing hormone agonists do not require preparation.^4^ Over half the patients referred engaged in fertility preservation methods, emphasizing the high interest in referrals. While only 8.3% of patients were ultimately successful in conception, this may be in part explained by the aggressive nature of the cancer in our population as over half of these individuals were diagnosed stage III/IV. Prompt establishment with oncofertility providers will help to create a tailored plan and allow management of any potential reproductive complications.

In our study, all three individuals with pregnancies were able to deliver full-term children without complications. There are few other retrospective studies examining the effects of ICIs on fertility. Mittra et al reported nine incidental pregnancies that occurred during National Cancer Institute sponsored clinical trials for ICI therapy.^5^ Of the 7 who proceeded with pregnancy, 6 carried their children to term and one developed preeclampsia resulting in a premature live birth. Similarly, Harrold et al described six patients who delivered full-term pregnancies following ICI therapy, of which one had a first-trimester miscarriage with a subsequent pregnancy.^6^ One mother developed gestational diabetes and another had preeclampsia. Rhodes et al discussed 8 patients with advanced-stage melanoma with nine spontaneous conceptions—3 during ICI therapy and 6 afterward.^7^ There were 3 miscarriages, 1 elective termination, and 5 healthy term deliveries. These groups of patients demonstrate that conception and term pregnancies are possible during and after ICI therapy.

Blockade of the PD1-PDL1 axis leads to impaired ovulation, diminished ovarian reserve, and depletion of antral follicles in mice.^8^ Depletion of primordial follicles may have lasting effects on fertility. In human placental tissue, the PD1-PDL1 axis can promote regulatory T cell development and subsequently increase maternal-fetal tolerance.^1^ During fetal development, maternal IgG antibodies can pass through the placental interface, and thus it is hypothesized the ICI therapies can traverse as well.^9^ Blockade of this axis has been hypothesized to lead to increased rates of preeclampsia, gestational diabetes mellitus, peripartum cardiomyopathy, and pre-term birth. Given the possibility of reduced fertility, it is essential that patients are counseled regarding fertility planning prior to ICIs initiation.

Limitations of this study include the concurrent or sequential use of chemotherapy, small sample size, and the retrospective nature of the study, potentially resulting in biases in patient selection, missing data, and fertility rates. Our study adds to the literature of successful pregnancy and live births following ICI therapy. As ICI use increases, prospective studies on its impact on reproductive outcomes are necessary. Future studies should incorporate tests, such as Anti-Mullerian hormone, a marker correlated with antral follicle count and can been used as a surrogate measure of ovarian reserve. Evaluation of the pituitary-gonadal axis would be prudent as hypogonadism and hypophysitis are possible immune related adverse events. In addition, there is a need for increased collaboration amongst medical oncology and oncofertility providers to increase awareness and expedite referrals to improve utilization of fertility preservation strategies.

In summary, successful conception following treatment with immunotherapy is possible. Larger multi-institutional studies are needed to further delineate the long-term effect of ICIs on ovarian function and reproduction.

## Data Availability

The data underlying this article will be shared on reasonable request to the corresponding author.
